# CD30 Expression in Neoplastic Mast Cells and the Presence of CD30+, CD3+, and PAX‐5+ Tumour‐Infiltrating Lymphocytes as Prognostic Markers in Canine Cutaneous Mast Cell Tumours

**DOI:** 10.1111/vco.70058

**Published:** 2026-02-25

**Authors:** Fernanda Ramalho Ramos, Janayna Maria Parente Serafim, Bethânia Almeida Gouveia, Pedro Luiz Porfirio Xavier, Juliano Rodrigues Sangalli, Heidge Fukumasu, Ricardo de Francisco Strefezzi

**Affiliations:** ^1^ Laboratory of Comparative and Translational Oncology, Department of Veterinary Medicine School of Animal Science and Food Engineering, University of São Paulo Pirassununga Brazil; ^2^ Department of Pathology School of Veterinary Medicine and Animal Science, University of São Paulo São Paulo Brazil; ^3^ Department of Veterinary Medicine School of Animal Science and Food Engineering, University of São Paulo Pirassununga Brazil

**Keywords:** cancer, immunohistochemistry, mast cells, prognosis, TNFRSF8

## Abstract

Canine cutaneous mast cell tumour (MCT) is the most common skin neoplasm in dogs, with histopathology serving as both the diagnostic and primary prognostic tool. However, identifying reliable biomarkers is essential for improving clinical decision‐making. CD30, a member of the TNF receptor superfamily, is well‐characterised in human haematopoietic malignancies. However, its role in canine MCTs remains unclear. This study aimed to evaluate CD30 expression in neoplastic mast cells and tumour‐infiltrating lymphocytes (TILs), and to assess its prognostic significance in canine cutaneous MCTs. Immunohistochemical analysis of CD30, CD3, and PAX‐5 was performed on 53 samples, and RNA sequencing was conducted to assess CD30 transcript levels in 17 cases. CD30 was detected in 94.6% of neoplastic mast cells, with strong staining observed in 35 cases and weak staining in 18. Strong CD30 expression (*p* = 0.0051), CD30+ TIL counts (*p* = 0.0105) and the diffuse infiltrate distribution pattern of CD3+ TILs (*p* = 0.0497) were associated with shorter post‐surgical survival. RNA sequencing confirmed higher CD30 (TNFRSF8) gene expression levels in high‐risk tumours (logFC = 2.3182; FDR = 0.0405). These findings suggest that CD30 is a promising biomarker with potential prognostic value in canine cutaneous MCTs.

## 1 Introduction

Canine cutaneous mast cell tumour (MCT) is the most common round cell neoplasm in dogs, accounting for up to 21% of all skin malignancies [1]. Histopathology remains the gold standard for diagnosis and prognostic evaluation, primarily through established grading systems, such as those proposed by Patnaik et al. [2] and Kiupel et al. [3] Nonetheless, given the biological heterogeneity of MCTs, identifying additional prognostic markers is crucial to guide therapeutic decisions and enhance clinical outcomes [4].

CD30 (Ki‐1 or TNFRSF8) is a transmembrane receptor from the Tumour Necrosis Factor (TNF) superfamily [5, 6, 7] that activates NF‐κB signalling, promoting pleiotropic gene regulation, cell proliferation, and anti‐apoptotic effects [8, 9]. First described in Hodgkin and Reed‐Sternberg cells in classical Hodgkin Lymphoma [6, 7], CD30 is physiologically expressed in a subset of activated CD4+ and CD8+ T cells, primarily involved in Th2‐type immune responses, as well as in activated B cells, NK cells, and dendritic cells [10, 11, 12].

In human oncology, CD30 is recognised as a marker of lymphoid neoplasms, including Hodgkin lymphoma and anaplastic large cell lymphoma [13], and is aberrantly expressed in neoplastic mast cells in systemic mastocytosis [14]. CD30 expression is associated with disease aggressiveness. Aggressive systemic mastocytosis and mast cell leukaemia display stronger CD30 staining intensity and a higher proportion of CD30+ mast cells [15], whereas cutaneous mastocytosis and indolent systemic mastocytosis exhibit detectable but comparatively lower CD30 expression [16, 17].

In dogs, CD30 expression has been described in anaplastic large cell lymphoma [18], pulmonary lymphomatoid granulomatosis [19], and more recently in neoplastic mast cells [20]. However, its diagnostic or prognostic significance in canine MCTs remains to be determined [21]. Furthermore, CD30 may influence the tumour microenvironment (TME) by promoting the release of immune mediators such as IL‐8, MIP‐1, CCL3, CCL5, and enhancing CXCL12–CXCR4 axis activity, thereby contributing to a Th1/Th2 response imbalance [22, 23, 24, 25].

TILs have previously been evaluated in canine cutaneous and subcutaneous MCTs, as demonstrated by Bertola et al. [26], who analysed the tumour immune microenvironment using Iba1 (macrophages), CD20 (B cells), CD3 (T cells), and FoxP3 (regulatory T cells). Their findings indicated that cutaneous and subcutaneous MCTs exhibit distinct immune profiles, and that T cell infiltration may help prevent nodal metastatic spread in cutaneous MCTs.

This study aimed to explore the prognostic significance of CD30 expression and the presence of CD30+, CD3+ (T cells), and PAX‐5+ (B cells) Tumour‐Infiltrating Lymphocytes (TILs) in canine cutaneous MCTs in relation to the histological grades, post‐surgical survival, tumour‐related mortality, and other well‐established prognostic markers (KIT and Ki‐67).

## 2 Materials and Methods

### 2.1 Samples

Fifty‐three formalin‐fixed, paraffin‐embedded samples of canine cutaneous mast cell tumours (MCTs) from 53 dogs were selected from the Tumour Bank of the Laboratory of Comparative and Translational Oncology (LOCT) at the Faculty of Animal Science and Food Engineering, University of São Paulo (FZEA‐USP), Brazil. The Ethics Committee for Animal Use of the FZEA‐USP approved the research under the protocol #7498190221. Cell line validation statement: no cell line was used in this study.

Clinical data were obtained from medical records and interviews with veterinarians or owners, including sex, age, breed, tumour site, lesion onset, date of surgery, local recurrence, lymph node metastasis (confirmed by histopathology), survival time, adjuvant chemotherapy, and cause of death. In dogs with multiple nodules, the tumour with the highest histological grade was analysed and considered in the survival analysis. The minimum post‐surgical follow‐up for censored cases was 180 days, and dogs that were treated with either neoadjuvant or adjuvant chemotherapy were excluded from the study.

### 2.2 Histological Processing and Analysis

Samples were fixed in 10% formalin for 48 h and embedded in paraffin. Tumour margins were evaluated using the radial sectioning technique (perpendicular margins) [27]. Four‐μm sections were stained with haematoxylin and eosin (HE) for the diagnosis and grading, as described by Patnaik et al. [2] and Kiupel et al. [3] by one observer (RFS). Mitotic figures were counted in a total area of 2.37 mm^2^.

### 2.3 Western Blot

To validate CD30 antibody specificity, SDS‐PAGE was performed on three frozen MCT samples (M1 and M2: grade II/high‐grade; M3: grade III/high‐grade) using human and canine lymph nodes as positive controls.

Protein was extracted from approximately 30 mg of each tissue using RIPA buffer, and 10 μg total protein was mixed with Laemmli buffer, boiled, and separated on 4%–20% SDS‐PAGE gels (100 V, 90 min) alongside a protein ladder (Precision Plus Protein All Blue, Bio‐Rad).

Proteins were transferred to PVDF membranes (Bio‐Rad) using a semi‐dry system (25 V, 3 min). Membranes were blocked with 3% BSA in TBS‐T for 1 h at room temperature, then incubated overnight at 4°C with CD30 (clone Ber‐H2, IR602, Dako, ready‐to‐use) diluted 1:2000 and mouse anti‐*β*‐actin HRP‐conjugated antibody (Sigma‐Aldrich, A3854) diluted 1:100 000.

After washing, membranes were incubated for 1 h with HRP‐conjugated anti‐mouse secondary antibody (Sigma‐Aldrich, A9044) diluted 1:10 000 in 1% BSA/TBS‐T. Detection was performed using Clarity Western ECL Substrate (Bio‐Rad) and visualised with the ChemiDoc MP Imaging System (Bio‐Rad). All experiments were performed in duplicate.

### 2.4 Immunohistochemistry

Sections were adhered to silanised slides, deparaffinised, and subjected to antigen retrieval in Tris‐EDTA (pH 9.0, 95°C, 25 min). Endogenous peroxidase was blocked with 6% hydrogen peroxide (CD30, CD3, PAX‐5) or Peroxidase Block reagent (Leica) (KIT, Ki‐67), both for 20 min at room temperature.

To prevent non‐specific binding, slides were incubated with poly‐l‐lysine (for CD30), 10% skim milk (for CD3 and PAX‐5), or with Protein Block reagent (Leica) for 5 min (for KIT and Ki‐67), at room temperature. Primary antibodies anti‐CD30 (clone Ber‐H2, Dako), anti‐CD3 (Dako, ready‐to‐use), anti‐PAX‐5 (clone DAK‐PAX5, Dako, ready‐to‐use), anti‐KIT (Dako, 1:600), and anti‐Ki‐67 (MIB‐1, Dako, 1:50) were applied overnight (4°C).

Detection was performed using the Novolink Polymer Detection System (Leica). The reactions were visualised using HRP Magenta (Dako) for CD30 and 3,3′‐diaminobenzidine (DAB; 1:50 dilution, Leica) for the other markers. Slides were counterstained with Harris haematoxylin. Canine lymph node (for CD30, CD3, and PAX‐5), skin (for Ki‐67), and MCT (for KIT) were used as positive controls. Negative controls were prepared by omitting the primary antibody and replacing it with PBS containing 1% bovine serum albumin (BSA).

### 2.5 Immunolabelling Analysis

All markers were evaluated using the 40× objective on an optical microscope (Leica DM500 + ICC50HD camera, 0.08 mm^2^ per image, total area = 0.4 mm^2^). CD30 expression was evaluated in five images from random fields per slide by manually counting CD30+ and CD30‐ mast cells (to determine the percentage of positive mast cells) and CD30+ lymphocytes (CD30+ TIL count). CD30 intensity was also classified as weak or strong, and the localisation was categorised as cytoplasmic or cytoplasmic + nuclear. CD3+ and PAX‐5+ TILs were manually counted in five hotspot fields in the intratumoral area. TILs distribution was categorised as diffuse infiltrate, aggregates (focal/multifocal clusters), or absent. KIT expression was evaluated according to Kiupel et al. [28] For Ki‐67, five hotspot fields were selected, and the Ki‐67 index was calculated as the percentage of positive nuclei by manually counting positive and negative mast cells. Tumours were subsequently categorised into low‐ and high‐proliferative‐index groups using a cut‐off value of 1.8%, as previously established [29]. All counts were performed using the ImageJ software (NIH, USA).

### 2.6 RNA‐Seq

RNA‐seq data from an additional 17 canine MCTs from a previous study of our research group [30] were analysed for TNFRSF8 (CD30) gene expression. MCTs were classified into high‐ or low‐risk groups using a score based on the histological grade, post‐surgical survival, Ki‐67 index, and tumour‐related death. Gene expression was estimated by read counts using HTseq and normalised as counts per million reads (CPM) [30]. Differential expression (DE) was performed using the EdgeR package on the R environment, based on negative binomial distribution [31]. Benjamini‐Hochberg procedure was used to control the false discovery rate (FDR), and FDR ≤ 0.01 and log‐fold change (LogFC) > 1 were considered differentially expressed (DE).

### 2.7 Statistical Analysis

CD30 expression (percentage of positive cells, CD30 intensity and CD30+ TIL counts) and CD3+ or PAX‐5+ TIL counts were assessed in relation to tumour grade 2‐tier classification [3] and tumour‐related mortality using either Mann–Whitney or unpaired *t*‐tests, depending on data distribution. Fisher's exact test was used to assess the associations between CD3+ and PAX‐5+ TILs distribution patterns and histological grades [3]. A Receiver‐Operating Characteristic (ROC) curve was plotted to establish a cut‐off for CD30+ TIL counts. Survival analyses were performed using the Kaplan–Meier method, followed by Mantel‐Cox/log‐rank and Gehan‐Breslow‐Wilcoxon tests. Dogs that died due to development of severe paraneoplastic syndromes (such as severe haemorrhage and anaphylactic reactions) and/or indication of euthanasia due to MCT by the veterinarian were considered deaths due to the disease for statistical analysis purposes. Pearson's correlation test was used to assess correlations between CD30 + TIL and CD3+ TIL, CD30+ TIL and PAX‐5+ TIL, and CD3+ and PAX‐5+ TIL counts. Associations between KIT expression patterns (KIT1, KIT2 and KIT3) and TILs counts were verified with one‐way ANOVA or chi‐square test for trend for CD30 staining intensity. Statistical analyses were performed using the GraphPad Prism software (GraphPad Inc., version 9.5.1 for MacOS, USA). The significance level was set at 5%.

## 3 Results

### 3.1 Clinical and Epidemiological Data

This study included 53 cases of canine cutaneous MCTs. The mean age was 10 years and 28 dogs were male (52.8%). Mixed‐breed dogs were most frequent (18/53; 34.0%), followed by Pit Bull (7/53; 13.2%), Labrador Retriever (6/53; 11.3%), and Boxer (5/53; 9.4%). All animals underwent surgical excision of the tumour, with wide margins (2–3 cm) [32], aiming for complete resection. Tumour location was recorded in 51 cases, most frequently on the limbs (22/51; 43.1%) and thorax (12/51; 23.5%). Twenty‐two (43.1%) dogs were alive at the end of the study, 21 (41.2%) died due to the MCT, and 8 (15.7%) died from other causes. Mean post‐surgical survival was 383.2 days (range: 4–1826 days). Local recurrence was reported in 12/53 cases (22.6%), and lymph node status in 38 cases. Nevertheless, only 13 cases had available lymph node tissues for the histological confirmation, with 3 presenting overt metastasis (3/13; 23.1%) and 7 presenting “early metastasis” [33].

### 3.2 Histopathological Analysis

According to the classification by Patnaik et al. [2], three of the tumours were graded as grade I (5.7%), 41 as grade II (77.3%), and nine as grade III (17.0%). Based on the Kiupel et al. [3] system, 35 (66.0%) were classified as low‐grade and 18 (34.0%) as high‐grade. The average mitotic count was 6 mitoses in 2.37 mm^2^ per sample, ranging from 0 to 28.

### 3.3 Western Blot

**FIGURE 1:**
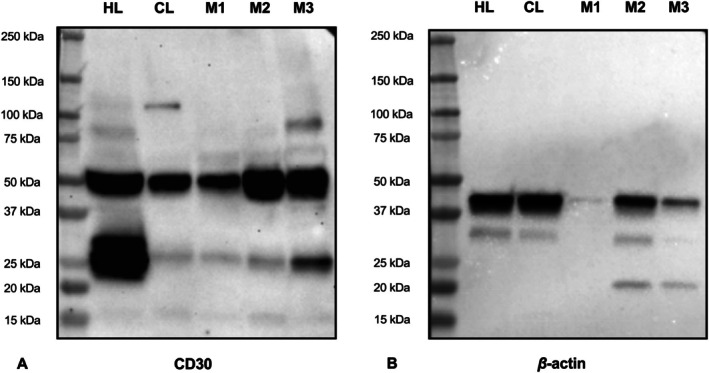
(A) Western blot analysis using the anti‐CD30 monoclonal antibody (clone Ber‐H2; IR602, Dako) in samples of human lymph node (HL), canine lymph node (CL), high‐grade grade II MCTs (M1 and M2), and high‐grade, grade III MCT (M3). (B) β‐Actin detection as a loading control.

Western blot analysis was used to investigate CD30 protein expression in human lymph node (HL), canine lymph node (CL), and canine cutaneous MCT samples. A faint band above 100 kDa (~120 kDa) was detected in the CL sample, and discrete bands below 100 kDa (~90 kDa) were observed in the HL and MCT sample M3. A strong band was detected in all samples at approximately 50 kDa (~57 kDa), and additional bands around 25 kDa were also observed, with greater intensity in the HL sample (Figure 1A). *β*‐actin (~42 kDa) was used as an internal loading control to verify the uniformity of protein loading across gel lanes. Consistent detection of *β*‐actin bands in all samples confirmed the homogeneity in total protein quantification and application (Figure 1B).

### 3.4 Immunohistochemical Analysis

**FIGURE 2:**
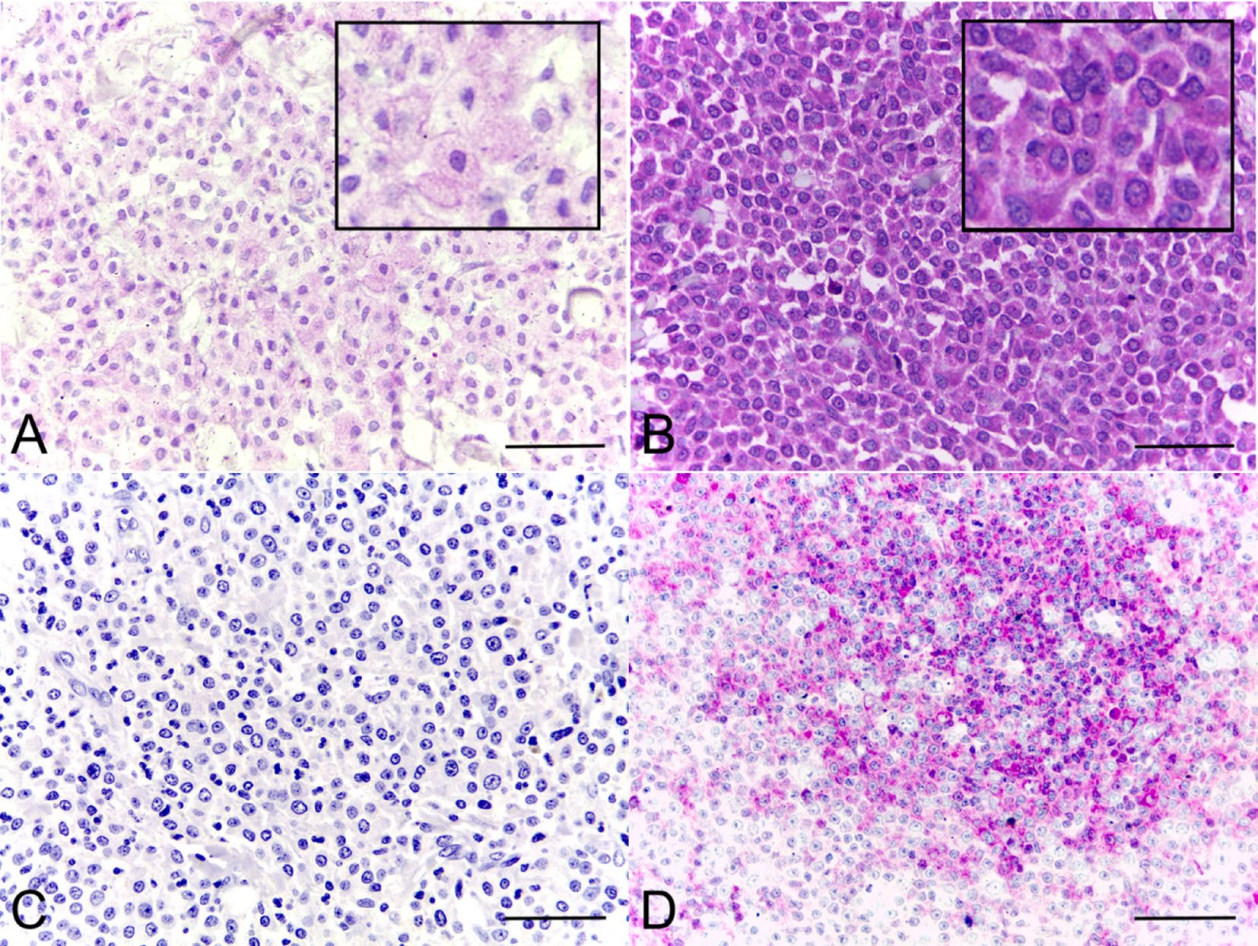
CD30 expression in canine cutaneous MCTs. (A) Weak CD30 immunolabelling in neoplastic mast cells (40× and 100×). (B) Strong CD30 immunolabelling in neoplastic mast cells (40× and 100×). (C) Negative control (canine MCT), obtained by omission of the primary antibody, showing absence of CD30 staining. (D) Positive control (canine lymph node), demonstrating CD30+ lymphocytes. Immunohistochemistry, magenta chromogen, counterstained with Harris' haematoxylin. Bar = 50 μm.

**FIGURE 3:**
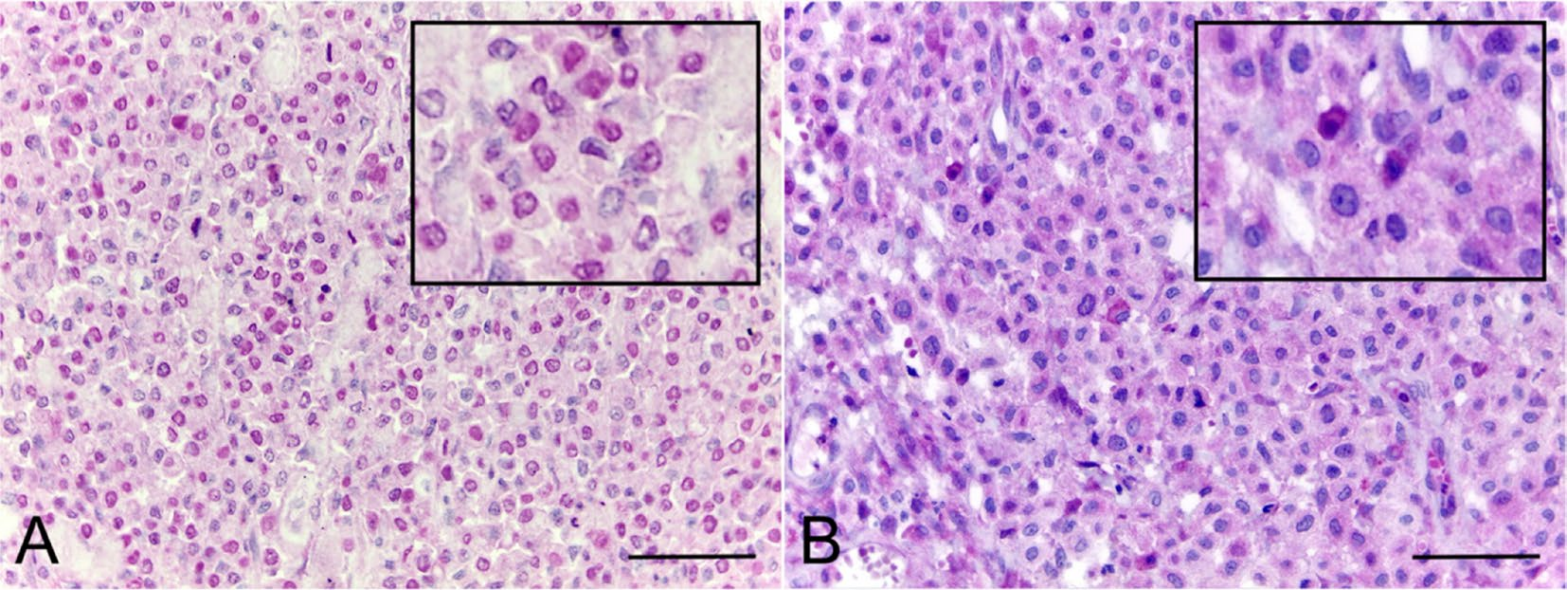
CD30 expression in canine cutaneous MCTs. (A) CD30 immunolabelling in neoplastic mast cells shows predominantly cytoplasmic staining, with approximately 75% of positive nuclei (inset shows positive nuclei). (B) CD30+ neoplastic mast cells arranged in sheets with strongly stained CD30+ tumour‐infiltrating lymphocytes (TILs) (inset shows two positive lymphocytes). Immunohistochemistry, magenta chromogen, counterstained with Harris' haematoxylin. Bar = 50 μm.

CD30 expression was detected in all MCTs, with variations in staining intensity and the percentage of positive cells. The mean percentage of CD30+ mast cells was 94.63% (range: 47.33%–100%) and the majority of the tumours presented strong intensity (35/53; 66.0%; Figure 2; Table 1). Staining intensity did not vary within individual tumours. CD30 immunostaining was predominantly cytoplasmic in neoplastic mast cells, though 20 cases (37.7%) also showed nuclear staining (Figure 3A).

**TABLE 1:** Distribution of CD30 expression variables according to prognostic parameters in dogs with cutaneous mast cell tumours.

Parameter	*n*	Percentage of CD30+ mast cells (mean ± SD)	CD30 intensity		CD30 localisation		CD30+ TILs (mean ± SD and range)
			Weak	Strong	Cytoplasmic	Cytoplasmic + nuclear	
Histological grade							
Patnaik [2]							
Grade I	3	96.8 ± 4.2	1	2	1	2	25 ± 21.1 (1–38)
Grade II	41	93.7 ± 10.8	16	25	24	17	17 ± 19.4 (0–79)
Grade III	9	98.2 ± 3.1	1	8	8	1	33 ± 28.1 (3–68)
Kiupel [3]							
Low‐grade	35	94.2 ± 8.3	14	21	19	16	15 ± 19.5 (0–79)
High‐grade	18	95.4 ± 12.2	4	14	14	4	23 ± 26.3 (0–68)
Mitotic count							
< 7/2.37 mm^2^	35	94.3 ± 8.3	13	22	19	16	12 ± 17.4 (0–63)
≥ 7/2.37 mm^2^	18	95.3 ± 12.2	5	13	14	4	28 ± 27.0 (0–79)
KIT pattern							
I	20	94.0 ± 9.4	6	14	11	9	17 ± 23.3 (0–79)
II	21	97.3 ± 2.9	8	13	15	6	15 ± 20.4 (0–68)
III	5	88.7 ± 23.1	2	3	3	2	23 ± 23.0 (4–55)
Ki67 index							
< 1.8%	10	92.1 ± 8.0	5	5	7	3	14 ± 24.5 (0–79)
≥ 1.8%	40	95.0 ± 10.4	12	28	24	16	19 ± 22.4 (0–68)
Mortality							
Alive	22	94.3 ± 8.6	9	13	11	11	8 ± 12.0 (0–43)
Dead	8	93.5 ± 9.6	4	4	4	4	20 ± 26.6 (0–79)
DDD	21	95.0 ± 11.5	3	18	16	5	21 ± 25.6 (0–68)

Abbreviations: DDD, dead due to the disease; HPF, high‐power field.

**FIGURE 4:**
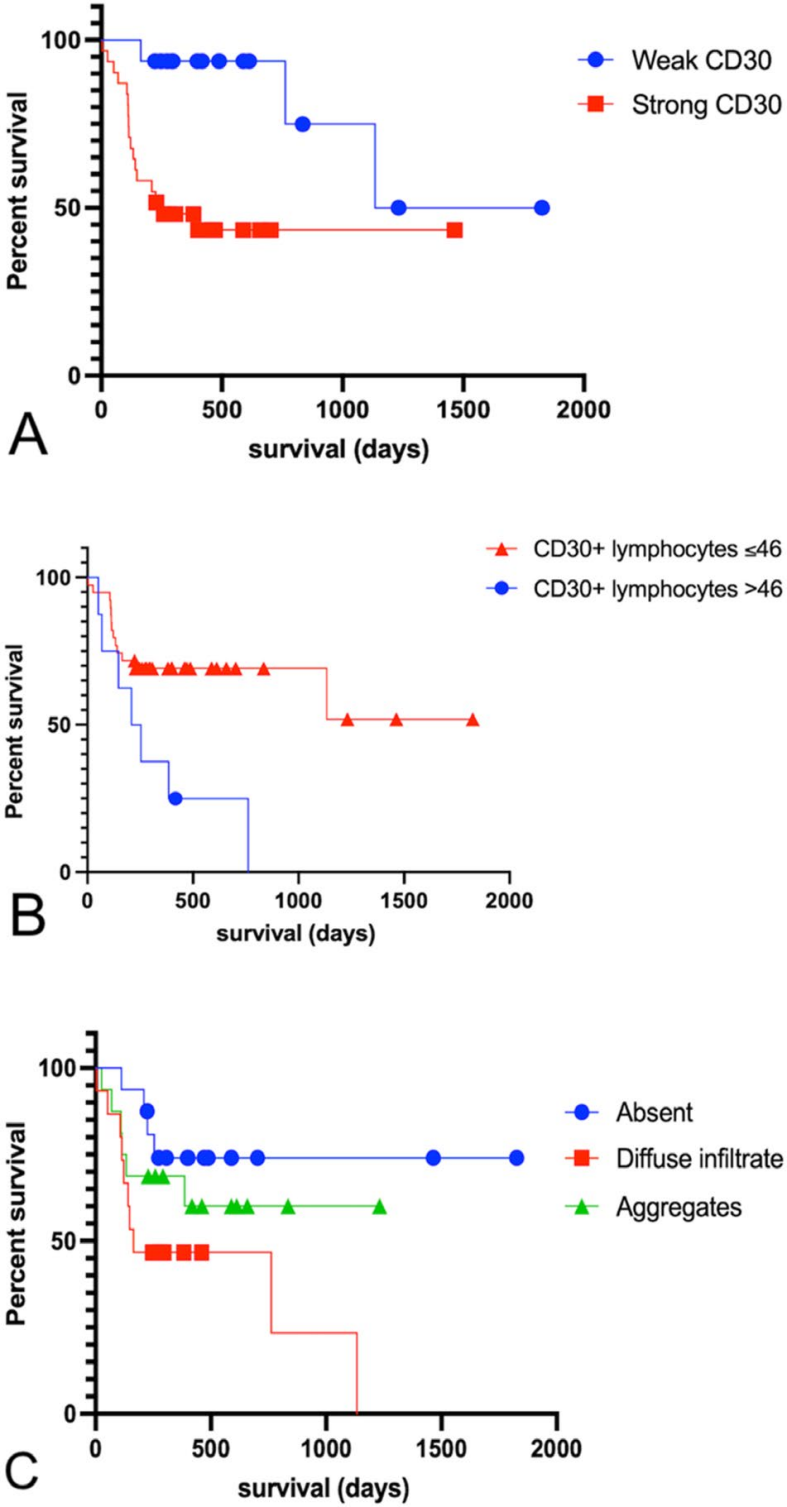
Survival curves of the dogs based on (A) the intensity of CD30 immunostaining in neoplastic mast cells (*p* = 0.0051; χ^2^ = 7.838; hazard ratio = 4.674; median survival for strong CD30 expression = 254 days; weak CD30 expression = 1480 days); (B) the CD30+ TIL counts in 2.37 mm^2^ (*p* = 0.0105; χ^2^ = 6.546; hazard ratio = 3.101; median survival for CD30+ Lymphocytes > 46 = 231.5 days); and (C) the distribution pattern of CD3+ TILs (*p* = 0.0497; χ^2^ = 5.689; hazard ratio = 3.692; median survival for diffuse infiltrate = 164 days). Kaplan–Meier survival analysis followed by Mantel‐Cox/log‐rank test. Dogs that died from other causes or were alive at the end of the study were censored (dots).

No significant differences were found in the percentage of CD30+ mast cells (*p* = 0.6697) or staining intensity (*p* = 0.0638) between low‐ and high‐grade MCTs. CD30 staining intensity was also not associated with mitotic count (*p* = 0.1397). Strong CD30 expression in mast cells was associated with shorter post‐surgical survival (*p* = 0.0051; χ^2^ = 7.838; hazard ratio = 4.674; median survival for strong CD30 staining = 254 days; weak CD30 expression = 1480 days; Figure 4A). Staining localisation (cytoplasmic vs. cytoplasmic + nuclear) was not associated with histological grade (*p* = 0.1368) or survival (*p* = 0.2289).

The mean CD30+ TIL count amongst all cases was 18 cells (range: 0–79 in 0.4 mm^2^; Table 1; Figure 3B). CD30+ TIL counts did not differ between low‐ and high‐grade MCTs (*p* = 0.1832). However, dogs that died due to the MCT showed higher CD30+ TIL counts (*p* = 0.0148). A cut‐off of 46 CD30+ TILs was identified via ROC curve analysis (AUC = 0.7028; *p* = 0.0185), and dogs with > 46 CD30+ TILs had a shorter survival (*p* = 0.0105; χ^2^ = 6.546; hazard ratio = 3.101; median survival for CD30+ lymphocytes > 46 = 231.5 days; Figure 4B).

**FIGURE 5:**
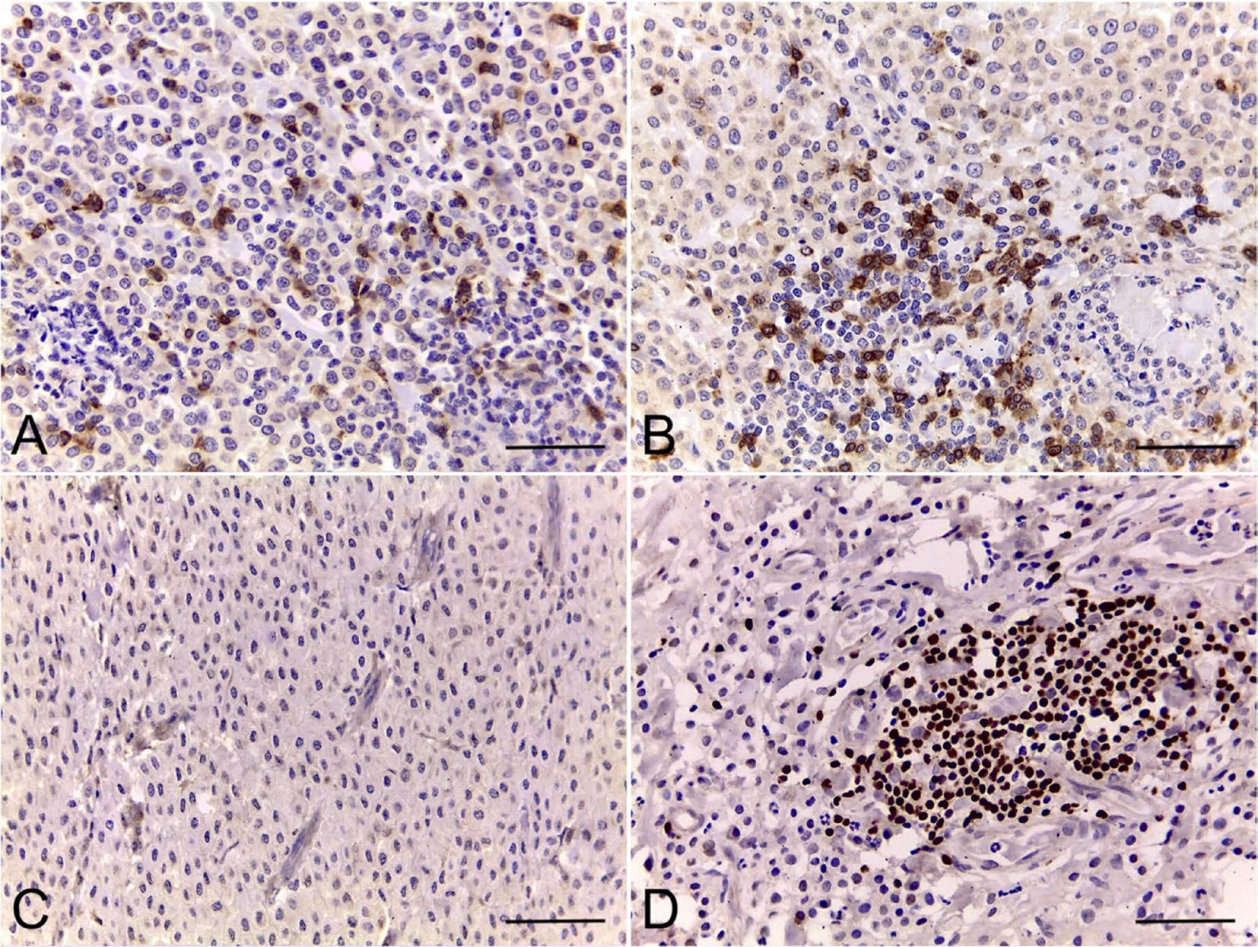
Photomicrographs of canine cutaneous MCTs showing the expression of CD3 and PAX‐5 in TILs. CD3 immunolabelling showing (A) diffusely distributed intratumoral T lymphocytes and (B) intratumoral T lymphocytes arranged in aggregates. PAX‐5 immunolabelling representing (C) absent pattern and (D) aggregates pattern. Immunohistochemistry, DAB chromogen, counterstained with Harris' haematoxylin. Bar = 50 μm.

CD3 expression was cytoplasmic and CD3+ TILs were absent in 16/53 (30.2%) of the samples. The diffuse infiltrate distribution pattern was observed in 18/53 (34.0%) and aggregates in 19/53 (35.8%) of the cases (Figure 5). The mean CD3+ TIL count was 95 (range: 0–969). PAX‐5 staining was nuclear. No cases exhibited a diffuse infiltrate pattern, whilst the PAX‐5 TILs were absent in 39/53 (73.6%) or aggregates in 14/53 (26.4%; Figure 5). The mean PAX‐5 TIL count was 103 (range: 0–2100). The aggregates pattern was primarily perivascular.

The CD3+ TIL count and distribution patterns were not significantly different between low‐ and high‐grade MCTs (*p* = 0.2401 and *p* = 0.4784, respectively). Although the presence of CD3+ TILs was not associated with survival (*p* = 0.0680), the diffuse infiltrate pattern was an indicator of shorter survival (*p* = 0.0497; χ^2^ = 5.689; hazard ratio = 3.692; median survival for diffuse infiltrate = 164 days; Figure 4C), especially in comparison with the absent pattern (*p* = 0.0171; χ^2^ = 5.689; hazard ratio = 3.692; median survival for diffuse infiltrate pattern = 164 days). PAX‐5+ TIL count and distribution were not associated with tumour grade (*p* = 0.8018 and *p* = 0.3329, respectively), nor survival (*p* = 0.3155).

CD30+ and CD3+ TIL counts were correlated (*p* = 0.0440; *r* = 0.2779), as well as CD30+ and PAX‐5+ (*p* = 0.0036; *r* = 0.3925), and CD3+ and PAX‐5+ TIL counts (*p* < 0.0001; *r* = 0.7011).

**FIGURE 6:**
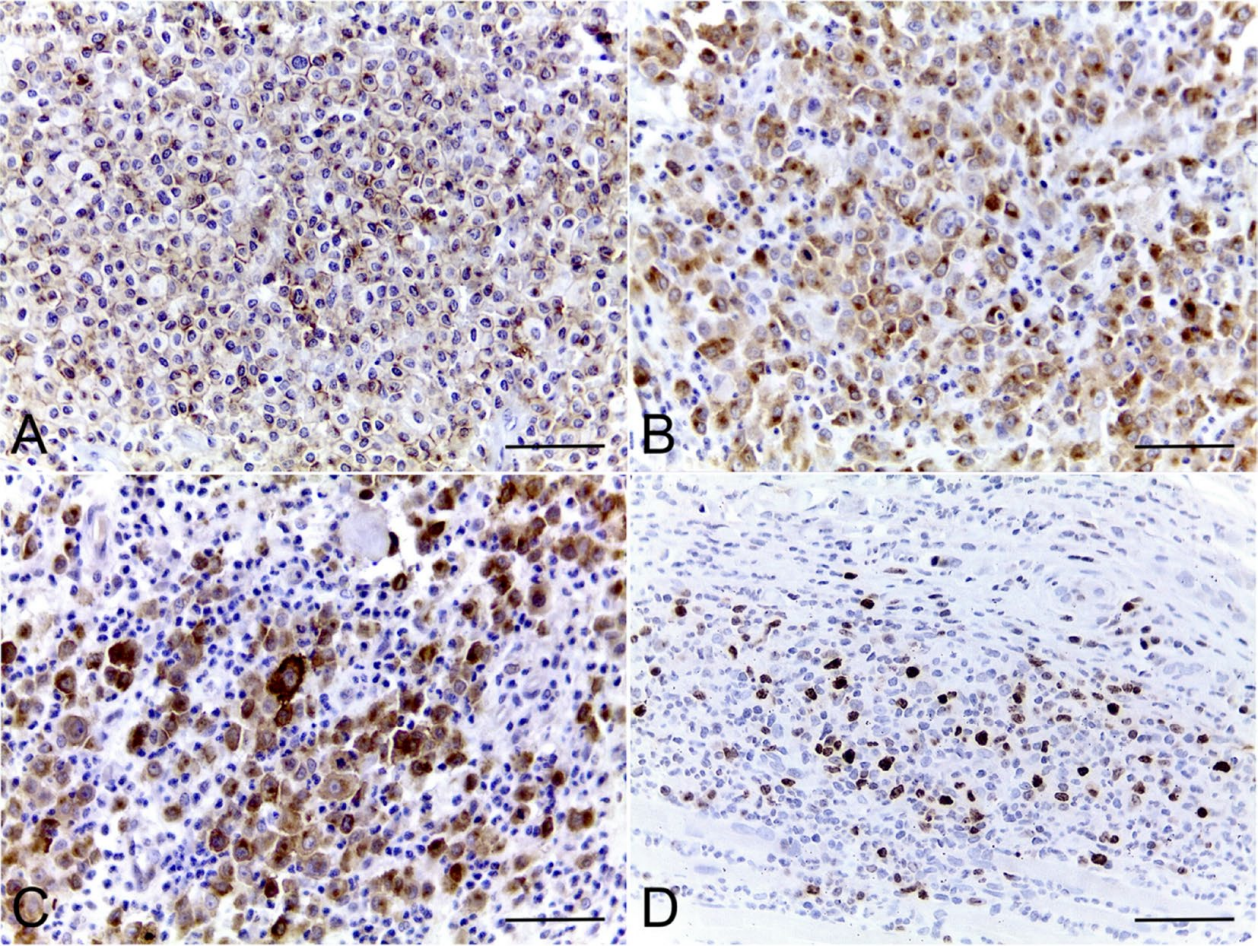
Photomicrographs of canine cutaneous MCTs showing (A) KIT pattern 1 (membranous staining), (B) KIT pattern 2 (focal cytoplasmic staining), (C) KIT pattern 3 (diffuse cytoplasmic staining), and (D) nuclear Ki‐67 expression. Immunohistochemistry, DAB chromogen, counterstained with Harris' haematoxylin, 40× objective.

KIT expression was evaluated in 46 of the MCT samples (Figure 6 and Table 2). No associations were found between KIT pattern and CD30 intensity (*p* = 0.5687) or CD30+ (*p* = 0.7954), CD3+ (*p* = 0.7121), and PAX‐5+ TIL counts (*p* = 0.1938). Ki‐67 expression (Figure 6D) was evaluated in 50 samples. The mean Ki‐67 index was 6.1% (range: 0%–21%), with a mean of 4.4% in the low‐grade and 9.1% in high‐grade MCTs. Ki‐67 was correlated with mitotic count (*p* = 0.0093; *r* = 0.3679), but not associated with CD30 staining intensity (*p* = 0.5296), percentage of CD30+ mast cells (*p* = 0.1828; *r* = 0.1915), or the CD30+ TIL count (*p* = 0.9953; *r* < 0.001).

**TABLE 2:** Distribution and quantification of CD3+ and PAX‐5+ lymphocytes across prognostic parameters in dogs with cutaneous mast cell tumours.

Parameter	*n*	CD3+ lymphocyte count (mean ± SD and range)	CD3+ lymphocyte distribution pattern			PAX‐5+ lymphocyte count (mean ± SD and range)	PAX‐5+ lymphocyte distribution pattern		
			Ab	DI	Ag		Ab	DI	Ag
Histological grade									
Patnaik [2]									
Grade I	3	0 ± 0 (0–0)	3	0	0	16.3 ± 28.3 (0–49)	2	0	1
Grade II	41	86.5 ± 99.7 (0–320)	11	13	17	81 ± 327.3 (0–2063)	30	0	11
Grade III	9	167.8 ± 319.5 (0–969)	2	5	2	233.8 ± 699.8 (0–2100)	7	0	2
Kiupel [3]									
Low‐grade	35	77.0 ± 96.9 (0–292)	13	9	13	93.2 ± 353.1 (0–2063)	24	0	11
High‐grade	18	131.2 ± 235.0 (0–969)	3	9	6	122.9 ± 494.1 (0–2100)	15	0	3
Mitotic count									
< 7/2.37 mm^2^	35	62.5 ± 89.9 (0–292)	14	10	11	27.0 ± 76.4 (0–411)	26	0	9
≥ 7/2.37 mm^2^	18	159.5 ± 230.8 (0–969)	2	8	8	251.6 ± 668.7 (0–2100)	13	0	5
KIT pattern									
I	20	118.0 ± 219.7 (0–969)	6	5	9	238.9 ± 633.9 (0–2100)	13	0	7
II	21	75.1 ± 107.8 (0–320)	7	9	5	5.1 ± 23.6 (0–108)	20	0	1
III	5	92.8 ± 97.2 (0–224)	0	3	2	16.6 ± 31.6 (0–73)	2	0	3
Ki67 index									
< 1.8%	10	43.7 ± 75.0 (0–218)	4	3	3	25.3 ± 80.0 (0–253)	9	0	1
≥ 1.8%	40	112.1 ± 174.4 (0–969)	11	14	15	130.5 ± 459.1 (0–2100)	27	0	13
Mortality									
Alive	22	97.2 ± 107.7 (0–320)	8	4	10	117.6 ± 436.8 (0–2063)	14	0	8
Dead	8	44.5 ± 75.6 (0–218)	4	2	2	31.6 ± 89.4 (0–253)	7	0	1
DDD	21	108.0 ± 220.0 (0–969)	4	11	6	120.0 ± 462.4 (0–2100)	17	0	4

Abbreviations: Ab, absent; Ag, aggregates; DDD, dead due to the disease; DI, diffuse infiltrates; HPF, high‐power field.

### 3.5 Differential Gene Expression Analysis of TNFRSF8 (CD30)

**FIGURE 7:**
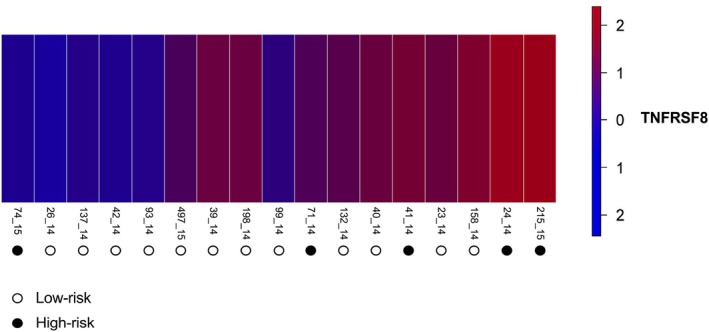
Heatmap of differential gene expression analysis. Higher TNFRSF8 gene expression is observed in MCT samples classified as high‐risk (*p* = 0.0212; FDR = 0.0424).

Differential expression of the TNFRSF8 gene was analysed in 17 canine cutaneous MCT samples. First, RNA‐Seq read counts were normalised to logCPM to account for differences in library size and to stabilise variance across genes with varying expression levels. The resulting logCPM values ranged from −1.62 to 6.89. High‐risk MCTs showed higher expression levels of TNFRSF8 than low‐risk MCTs (logFC = 2.3182, *p* = 0.0212; FDR = 0.0424; Table 3; Figure 7).

**TABLE 3:** Summary of TNFRSF8 (CD30) gene expression in low‐ and high‐risk canine cutaneous mast cell tumours.

Risk group	*n*	Mean log value	Min	Max
Low‐risk	12	1.92	−1.62	5.48
High‐risk	5	4.00	−1.06	6.89

## 4 Discussion

In the present study, we characterised CD30, CD3, and PAX‐5 expressions in canine cutaneous MCTs and investigated their potential as prognostic markers for the disease. All samples showed CD30+ mast cells, regardless of histological grade, and strong staining intensity was significantly associated with shorter post‐surgical survival. In human mast cell neoplasms, however, the prognostic value of CD30 is not entirely consistent across studies. Aggressive systemic mastocytosis and mast cell leukaemia have also been associated with increased CD30 staining intensity and a greater proportion of CD30+ mast cells [15]. Conversely, Silva et al. [34] reported stronger CD30 staining and a higher percentage of positive mast cells in less aggressive human mast cell neoplasms, suggesting that CD30 expression is not exclusive to more aggressive disease forms.

CD30 is abundantly expressed in neoplastic mast cells and in advanced forms of systemic mastocytosis in humans [13, 14], as well as canine MCTs [20]. The mature form of human CD30 is a type I transmembrane glycoprotein [9], with a molecular weight of 105–120 kDa, derived from a 90 kDa precursor [5], and a non‐glycosylated intracellular isoform of approximately 57 kDa [35]. This intracellular isoform has been described in the cytoplasm, nuclear pores, and nuclei [36, 37]. In our samples, immunostaining was predominantly cytoplasmic, though nuclei also showed positive labelling. Western blot analysis using an anti‐CD30 antibody revealed multiple protein isoforms. In canine lymph node, a band around 120 kDa was observed, consistent with the mature glycosylated form of CD30. In contrast, this band was not detected in human lymph node. A plausible explanation is that the human lymph node sample contained very few or no CD30‐expressing activated lymphocytes. CD30 expression in normal human lymph nodes is typically low or absent, with rare activated immunoblasts exhibiting restricted expression [5]. Consequently, the mature glycosylated isoform (~120 kDa) may have been below the detection threshold in this tissue.

In human lymph node and MCT sample 3, another band was detected, of approximately 90 kDa, probably corresponding to the precursor form of the protein. All samples also showed a strong band around 50 kDa, suggesting the presence of the 57 kDa intracellular, non‐glycosylated isoform [37], consistent with our immunohistochemical findings of cytoplasmic and occasional nuclear labelling, indicative of active intracellular CD30 isoforms.

In addition, a 25 kDa band was consistently detected across all Western blot samples. This band likely represents CD30 variant (CD30v), an isoform resulting from alternative splicing of the TNFRSF8 gene, which encodes a cytoplasmic form lacking the transmembrane domain, with an estimated molecular weight of 25 kDa [38, 39]. Although the functional role of CD30v remains poorly understood, it is known to be driven by a cryptic promoter within intron 10 of the TNFRSF8 gene, resulting in a ligand‐independent isoform that contains only the signalling domain [39]. In transformed human embryonic stem cell (hESC) models, CD30v is overexpressed and associated with increased cell survival and proliferation, primarily through activation of the NF‐κB pathway. There is also evidence of its nuclear localisation [40]. CD30v has been frequently identified in neoplastic myeloid and lymphoid human cells, often in association with TRAF family proteins [41].

Dong and Zhou [42] analysed 13 members of the TNFRSF gene family across 23 different human cancer types and found that high expression of TNFRSF1B, TNFRSF9, TNFRSF11A, TNFRSF8, TNFRSF13C, TNFRSF12A, LTBR, and RELT correlated with oncogene expression and poor overall survival. In our study, elevated TNFRSF8 (CD30) expression was associated with high‐risk MCTs.

TILs have been extensively studied, with increasing evidence of their role in cancer prognosis. Additionally, Bertola et al. [26] provided important insights into the tumour immune microenvironment of canine cutaneous and subcutaneous MCTs, showing that T cell infiltration might help prevent nodal metastatic spread in cutaneous MCTs. However, their panel included macrophages (Iba1), B cells (CD20), T cells (CD3), and regulatory T cells (FoxP3). The prognostic significance of specific T cell and B cell subsets remains not fully understood, highlighting the need for further studies like the present one.

We observed that the presence of CD30+ infiltrating lymphocytes was associated with MCT‐related mortality. Moreover, infiltration of more than 46 CD30+ TILs in 2.37 mm^2^ was an indicator of shorter post‐surgical survival. CD30 actively participates in shaping the TME [22], mediating interactions with CD30L that result in the release of immune mediators, such as IL‐8, MIP‐1α, MIP‐1β, CCL3, and CCL5, along with enhanced CXCL12 chemotactic activity and CXCR4 expression [23, 24]. CD30 signalling also promotes IFN‐γ, IL‐4, IL‐5, IL‐12p70, and IL‐12p40 secretion, modulating Th1/Th2 responses [25].

CD30 can also impair the functions of cytotoxic T cells and NK cells by downregulating effector molecules such as perforin, granzyme, and Fas ligand, thus reducing their anti‐tumour capacity. Additionally, CD30 signalling may suppress c‐Myc expression, a key regulator of cell proliferation and Fas ligand expression, thereby facilitating immune evasion [22].

To characterise the phenotypes of TILs, immunohistochemistry was performed for CD3 (T cells) and PAX‐5 (B cells). Costa et al. [43] found no significant differences in T‐cell infiltration between high‐ and low‐grade MCTs, consistent with our findings. Although T cell counts were not correlated with post‐surgical survival, the presence of diffuse infiltrate was associated with shorter survival compared to the absence of T cells. This contrasts with the findings of Yasumaru et al. [44], who observed that diffuse infiltrate and aggregates TIL patterns in canine oral melanomas were associated with longer survival.

T cells play a central role in anti‐tumour immunity, particularly cytotoxic T cells [45, 46]. However, the TME often impairs T‐cell infiltration and function, promoting T‐cell exhaustion and immune evasion [46]. In contrast, B‐cell infiltration in MCTs remains poorly investigated [26]. In our study, the presence, distribution and number of PAX‐5+ TILs were not associated with histological grade or survival. B cells can exhibit both pro‐ and anti‐tumour functions. Regulatory B cells secrete immunosuppressive cytokines that support tumour growth, whilst anti‐tumour B cells can inhibit neoplastic progression by producing tumour‐specific antibodies [22].

Cooperation between T and B cells is essential for robust, long‐lasting immune responses, including the development of immunological memory [47]. We observed positive correlations between CD30+, CD3+, and PAX‐5+ TIL counts. However, none of these markers were independently associated with patient post‐surgical survival or tumour‐related mortality. Due to technical limitations, we were unable to determine whether CD30+ lymphocytes were T or B cells, which would have provided further insight into the cellular context of CD30 expression.

Analysis of KIT expression revealed predominantly KIT‐1 and KIT‐2 patterns, consistent with previous reports [28]. CD30 staining intensity and lymphocyte infiltration were not correlated with KIT expression, suggesting that they are not associated. High‐grade MCTs showed high Ki‐67 indexes [29, 48, 49, 50], however, no correlations were found between Ki‐67 and the immune markers.

Despite the contributions of this study, some limitations must be acknowledged. The limited number of grade I tumours restricted our statistical comparisons to the Kiupel et al. [3] system. Although CD30 expression and TIL counts were associated with survival outcomes, they were not correlated with other established prognostic parameters such as Ki‐67, KIT expression, or histological grade, suggesting that CD30 may reflect distinct aspects of tumour biology or the immune microenvironment.

In addition, the overall number of cases included in this study was smaller than originally intended. This limitation resulted primarily from limited tissue availability and the exhaustion of some of the paraffin blocks. Consequently, not all immunohistochemical analyses could be performed in the same number of samples. Despite this constraint, the 53 cases analysed represent all eligible tumours with complete clinical, histopathological, and follow‐up data available during the study period, providing a consistent and representative dataset for evaluating the prognostic relevance of CD30 and tumour‐infiltrating lymphocytes in canine MCTs.

CD30 and immune marker expression were evaluated only by immunohistochemistry, without functional assays to explore downstream signalling or tumour‐immune system interactions. Future studies should include larger cohorts, integrated molecular analyses, and detailed immune profiling to clarify the immunobiological role of CD30 in canine cutaneous MCTs. Nevertheless, our findings enhance the understanding of CD30 expression in MCTs and support its potential prognostic relevance. We believe that the present work lays the foundation for future research exploring the mechanistic role of CD30 and its potential as a biomarker or therapeutic target in canine oncology.

## Funding

This work was supported by Fundação de Amparo à Pesquisa do Estado de São Paulo (2020/10582‐0, 2022/09378‐5, 2022/06305‐7, 2023/00296‐9, 2023/05099‐7) and Conselho Nacional de Desenvolvimento Científico e Tecnológico (303748/2021‐4).

## Conflicts of Interest

The authors declare no conflicts of interest.

## Data Availability

The data that support the findings of this study are available on request from the corresponding author. The data are not publicly available due to privacy or ethical restrictions.
